# Coupled-enzyme assay for MTAP activity in biological samples

**DOI:** 10.1016/j.ab.2026.116109

**Published:** 2026-03-11

**Authors:** Nord Gilaj, Andrew G. Wagner, Terence Li, Dior Dedushi, Roozbeh Eskandari, Chaoyuan Kuang, Vern L. Schramm

**Affiliations:** a Department of Biochemistry, Albert Einstein College of Medicine, Bronx, NY, 10461, United States; b Departments of Medicine, Oncology and Molecular Pharmacology, Albert Einstein College of Medicine, Bronx, NY, 10461, United States

**Keywords:** *In vivo* enzyme assay, Continuous enzyme assay, Adenine detection, Synthetic-lethal interactions, Methionine pathway

## Abstract

5′-Methylthioadenosine phosphorylase (MTAP) catalyzes the reversible phosphorolysis of 5′-methylthioadenosine (MTA), a product of polyamine synthesis. The resulting products, adenine and 5-methylthio-α-d-ribose 1-phosphate (MTR-1-P), are salvaged to the adenylate and methionine pathways. Homozygous deletion of MTAP occurs in approximately 15% of all human cancers due to its chromosomal proximity to the CDKN2A/B locus. Loss of MTAP creates a metabolic vulnerability to PRMT5 and MAT2A inhibition, resulting in a synthetic lethal interaction. Consequently, MTAP has emerged as a target for enzymatic inhibition to enable synthetic lethal strategies in the 85% of cancers that are MTAP^+/+^. Analysis of MTAP inhibitors in biological samples necessitates a robust assay for MTAP activity. Previous assays for MTAP activity have relied on radiolabeled substrates and chromatography, methods that are cumbersome for routine testing. We report a continuous assay suitable for biological samples that quantifies MTAP activity by coupling adenine deaminase, xanthine oxidase, and horseradish peroxidase. The method is validated using whole blood and by analyzing kinetic and inhibition constants with transition-state analogs, methylthio-DADMe-Immucillin-A (MTDIA) and *para*-chloro-phenylthio-DADMe-Immucillin-A (*p*ClPhTDIA). MTAP activity was also measured across a panel of cancer cell lines to determine intracellular MTAP levels and to complement whole-blood measurements. This approach enables real-time evaluation of MTAP function and drug response in μL volumes of lysate, providing a direct biological or clinical platform for tracking targeted MTAP therapies.

## 1. Introduction –

**Scheme 1.**
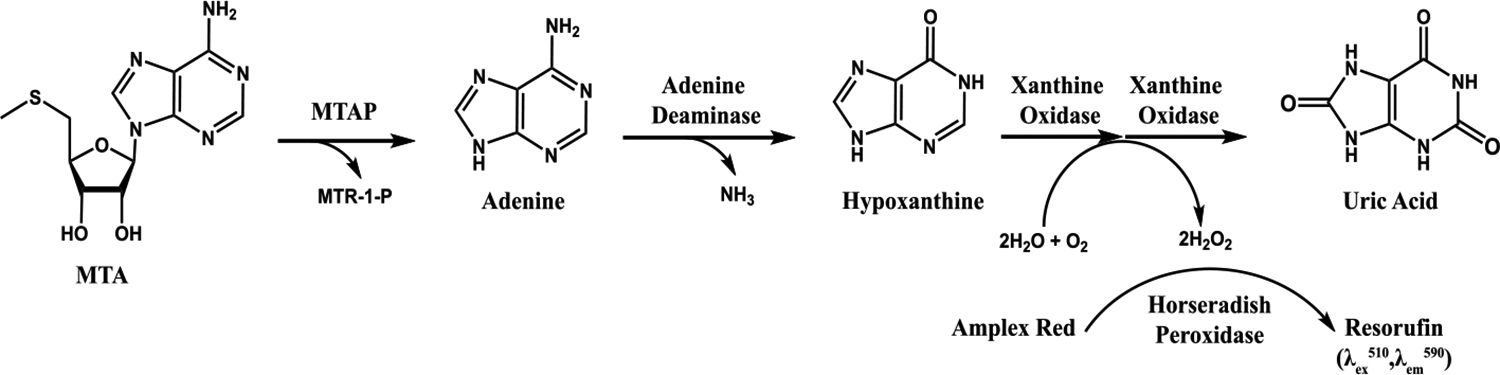
Coupled assay for sensitive detection of MTAP catalysis. MTA, 5′-methylthioadenosine; MTAP, 5′-methylthioadenosine phosphorylase; ADA, adenine deaminase; XanOx, xanthine oxidase; HRP, horseradish peroxidase.

In human metabolic pathways, 5′-methylthioadenosine phosphorylase (MTAP, EC:2.4.2.28) is the sole enzyme responsible for catalyzing the removal of 5′-methylthioadenosine (MTA), a product of propylamine group transfer from decarboxy-S-adenosyl-l-methionine (dcSAM) during polyamine biosynthesis [1,2]. The MTAP-catalyzed reaction produces adenine and 5-methylthio-α-d-ribose 1-phosphate (MTR-1-P) from MTA and phosphate (Scheme 1), thereby providing metabolites for adenine salvage through purine salvage pathways and enabling regeneration of methionine from MTR-1-P [3,4]. MTAP catalytic activity is essential to maintain low cellular concentrations of MTA, a metabolite that acts as a product inhibitor of both spermidine and spermine synthases (EC:2.5.1.16 and EC:2.5.1.22) and is a SAM-competitive inhibitor of protein arginine methyl transferase 5 (PRMT5, EC:2.1.1.320). Loss of MTAP activity causes the accumulation of MTA and the inhibition of downstream signaling pathways linked to PRMT5 activity [5,6]. Genetic deletion of the MTAP locus occurs in approximately 15% of cancers due to its proximity to the CDKN2A/B locus, making it one of the most frequently deleted genes in human cancers [7,8]. Synthetic lethal screens comparing MTAP^+/+^ and MTAP^−/−^ cancer cell lines have identified synthetic lethal interactions between MTAP and either methionine adenosyltransferase 2A (MAT2A, EC:2.5.1.6) or PRMT5 [9,10]. These deletions affect PRMT5 activity by combining MTA mediated inhibition with S-adenosyl-l-methionine (SAM) depletion. SAM is an essential substrate for PRMT5, and MAT2A functions as the SAM-synthetase. Anti-cancer agents against MAT2A or PRMT5 are under investigation in over a dozen clinical trials, specifically for those 15% of cancers that are MTAP^−/−^ [10–12]. Pharmacologic inhibition of MTAP activity mimics the MTAP^−/−^ phenotype and potentially extends these programs to the 85% of human cancers that are MTAP^+/+^. The transition state of human MTAP has been characterized through kinetic isotope effect (KIE) measurements and computational modeling, facilitating the rational design of potent transition state (TS) analog inhibitors [2,13,14]. Among these, the TS analog inhibitors methylthio-DADMe-Immucillin-A (MTDIA) and *para*--chloro-phenylthio-DADMe-Immucillin-A (*p*ClPhTDIA) exhibit kinetic inhibition constants (*K*_i_* values) of 86 and 10 pM, respectively, and are characterized by slow-onset, tight-binding [13]. MTDIA has shown promising anti-cancer activity in preclinical models, significantly reducing MTAP activity in the blood and tissues of treated mice and inducing synthetic lethal responses in tumors when combined with MAT2A inhibition [9,15].

MTAP is present in erythrocytes and represents the primary source of MTAP activity in whole blood [15]. Previous studies measuring MTAP activity in blood samples have used radiolabeled MTA, with reaction products analyzed by cellulose or silica TLC [16]. Although radiometric assays offer high sensitivity, they require extensive sample handling, radioactive materials, and multi-step processing, which limit their practicality [17]. Absorbance-based assays have also been used to measure MTAP activity in crude cell extracts, however, these approaches offer limited sensitivity and a narrow range, making them unsuitable for samples with low activity [18]. In contrast, the fluorescence-coupled assay in this study excels at high sensitivity, applicability to small sample volumes, and compatibility with high-throughput microplate formats, thus providing a practical and robust method for quantifying MTAP activity in both blood and cellular samples [17,19].

This MTAP assay is validated using steady-state kinetics and by inhibiting MTAP activity with transition state analogs. Complete inhibition of MTAP replicates the MTAP^−/−^ physiological state, and analysis of blood and tissues provides a framework for evaluating pharmacologic inhibition.

## 2. Materials and methods –

### 2.1. General information

All buffer components for purification and assay of MTAP activity were purchased from Sigma-Aldrich. Amplex Red (#10010469) was purchased from Cayman Chemical. Hydrogen peroxide (30%; #H1009), xanthine oxidase (#X2252), horseradish peroxidase (#P8125), and Triton X-100 (#T9284) were purchased from Sigma-Aldrich. Corning^™^ 96-Well Solid Black, Polystyrene Microplates (#3915) were purchased from Fisher Scientific (#07-200-590).

### 2.2. Protein expression and purification

Human MTAP (EC:2.4.2.28, *hs*MTAP) was expressed in *E. coli* BL21 (DE3) *E. coli* (NEB, C2527) and purified as previously described (plasmid available from Addgene #64077) [13,20]. *Pseudomonas aeruginosa* adenine deaminase (*Pa*ADA; UniProt Q9I6Y4) [20] was produced using a pET-28a (+) vector (GenScript) encoding an N-terminal His_6_ tag followed by a TEV protease cleavage site (expression plasmid submitted to Addgene, #250880). The construct was transformed into *E. coli* One Shot^™^ BL21 (DE3) pLysS cells (Thermo Fisher Scientific, Cat. #C606010). Single colonies were inoculated into 20 mL LB medium supplemented with 50 μg/mL kanamycin and 30 μg/mL chloramphenicol and cultured overnight at 37 °C. The resulting cultures were used to inoculate 1 L LB medium containing the same antibiotics, grown at 37 °C to an optical density of 0.5 at 600 nm before induction with 1 mM IPTG. After 20 h at 20 °C, cells were collected by centrifugation. Cell pellets were resuspended in 20 mM Tris-HCl buffer (pH 7.4) containing 500 mM NaCl, 10 mg/mL lysozyme, 0.1% Triton X-100, and 5 mM imidazole and lysed by sonication (10 min total, 10 s on/off cycles) on ice. The lysate was clarified by centrifugation at 18,000 rpm for 1 h and the supernatant was applied to a 5 mL HisTrap FF Ni–NTA affinity column (Cytiva). The column was washed twice with four column volumes of 20 mM Tris-HCl (pH 7.4) containing 500 mM NaCl and 50 mM imidazole. Bound *Pa*ADA was eluted using four column volumes of the same buffer containing 500 mM imidazole. Eluted fractions containing the target protein were pooled and concentrated using a 10 kDa molecular weight retention Amicon Ultra centrifugal filter (Millipore) and stored at −80 °C with 10% (v/v) added glycerol. Final *Pa*ADA isolated yield was 48 mg/L bacterial culture.

### 2.3. Cell culture of cancer cell lines

All cell lines originated from the American Type Culture Collection (ATCC), including HT-29 (ATCC HTB-38), HCT-116 (ATCC CCL-247), RKO (ATCC CRL-2577), SKOV3 (ATCC HTB-77), AsPC-1 (ATCC CRL-1682), and MIA PaCa-2 (ATCC CRL-1420). Cells were maintained in Gibco McCoy’s 5A medium (Thermo Fisher Scientific 16600082) supplemented with 10% fetal bovine serum (Thermo Fisher Scientific 16000069) and 1% penicillin–streptomycin (Cytiva SV30010) at 37 °C in a 5% CO_2_ incubator, using tissue-culture treated 10 cm dishes. MTAP^−/−^ HT-29 isogenic knockout clones were purchased from GenScript, and loss of MTAP expression was confirmed by Western blotting.

### 2.4. MTAP kinetic assays

Whole human blood was collected in EDTA tubes during phlebotomy for standard-of-care blood collection or from cancer patients and banked with the Montefiore Einstein Comprehensive Cancer Center Biobank (Albert Einstein College of Medicine IRB #2021–13730). De-identified whole blood was used for experiments in accordance with institutional guidelines and handled under approved biosafety and regulatory protocols (IRB #2025–16811). All assays were performed at 37 °C and initiated by the addition of purified recombinant *hs*MTAP, aliquots from lysed human blood, or cell lysates. Reaction wells of 96-well polystyrene plates contained a final volume of 100 μL. No-MTA and no-MTAP controls were included to account for background activity. Fluorescent steady-state kinetic assays were performed on a SpectraMax iD3 Microplate Reader (Molecular Devices) using 510 nm excitation and 590 nm emission wavelengths. Kinetic rate data were analyzed and fit using GraphPad Prism 10.

### 2.5. Optimized assay conditions

#### 2.5.1. Hydrogen peroxide standard curves

Reactions were performed in 100 mM K_2_HPO_4_ buffer at pH 7.4 containing 0.1 U/mL xanthine oxidase, 0.1 U/mL horseradish peroxidase, 0.5 μM adenine deaminase, and 40 μM Amplex Red. All reaction plates were incubated at 37 °C for 15 min prior to endpoint readout. Reactions without hydrogen peroxide served as controls and were used to correct for non-enzymatic background rates. Hydrogen peroxide concentrations were plotted against relative fluorescence units (RFU) and fitted by linear regression to generate standard curves.

**Fig. 1.**
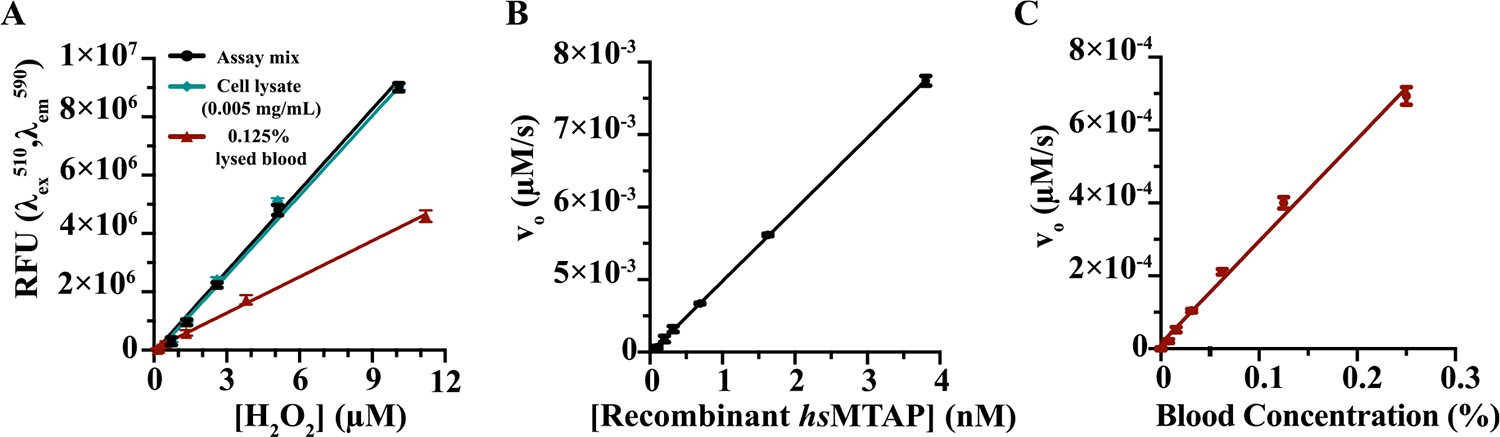
Quantitation of MTAP reaction conditions. **(A)** Standard curve of fluorescence response to hydrogen peroxide in assay mixture with 0.5 μg cell lysate/well or lysed blood from 0.125 μL whole blood. **(B)** Linear range for reaction rate as a function of recombinant human MTAP. **(C)** Reaction rate as a function of lysed blood concentration (e.g., 0.25% is 0.25 μL blood/100 μL assay mixture).

For recombinant *hs*MTAP reactions, 10 nM purified enzyme was added to each well with hydrogen peroxide concentrations ranging from 39 nM to 40 μM in two-fold increments. All assays using whole blood were diluted to 1.25% (v/v) in 100 mM K_2_HPO_4_ (pH 7.4) buffer containing 0.1% Triton-X 100 and incubated on ice for 30 min. Then, 10 μL of lysed blood was added to the reaction mixture to yield a final blood concentration of 0.125% (v/v). The effect of 10 μL of lysed blood on fluorescence was determined with hydrogen peroxide concentrations ranging from 4.5 nM to 100 μM in three-fold dilutions (Fig. 1C). The same H_2_O_2_ titration scheme was used to generate the standard curve in the presence of cancer cell lysates diluted to 0.005 mg/mL total protein, similar to the MTAP activity assays in Fig. 6A.

#### 2.5.2. Recombinant hsMTAP standard curve

Reactions contained 100 mM K_2_HPO_4_ (pH 7.4), 500 μM MTA, 0.1 U/mL horseradish peroxidase, 0.1 U/mL xanthine oxidase, 0.5 μM adenine deaminase, and 40 μM Amplex Red. Reactions were initiated with MTAP in three-fold increments from 1.7 pM to 100 nM. Initial rates from reactions were plotted against MTAP concentration and fitted by linear regression.

#### 2.5.3. MTAP activity in blood lysates

Assay mixtures contained 100 mM K_2_HPO_4_ (pH 7.4), 500 μM MTA, 0.1 U/mL horseradish peroxidase, 0.1 U/mL xanthine oxidase, 0.5 μM adenine deaminase, and 40 μM Amplex Red. Lysed blood was diluted to obtain assay well concentrations ranging from 0.04% to 10% (v/v) relative to whole blood. Reactions were initiated by the addition of 10 μL blood lysate. Initial reaction rates were calculated as in section 2.5.2.

### 2.6. MTAP kinetics from lysed human blood

MTAP activity in lysed blood was determined with MTA concentrations from 0.2 to 100 μM to establish Michaelis-Menten parameters. Assay mixtures contained 100 mM K_2_HPO_4_ (pH 7.4), 0.1 U/mL horseradish peroxidase, 0.1 U/mL xanthine oxidase, 0.5 μM adenine deaminase, and 40 μM Amplex Red. Background fluorescence from no-MTA controls was subtracted from all rates. The initial reaction rates were plotted against MTA concentration and fit to the Michaelis-Menten equation using GraphPad Prism to calculate *V*_Max_ and *K*_m_ values using the equation:

(eq. 1) $$v=\left(V_{\text{Max}}\left[\text{s}\right]\right)/\left(K_{m}+\left[\text{S}\right]\right)$$

where *v* is the initial reaction rate and [S] is the concentration of the MTA substrate.

### 2.7. MTAP inhibition assay

Reaction mixtures contained 100 mM K_2_HPO_4_ (pH 7.4) containing 1.5 mM MTA, 0.1 U/mL horseradish peroxidase, 0.1 U/mL xanthine oxidase, 0.5 μM adenine deaminase, and 40 μM Amplex Red. MTDIA or *p*ClPhTDIA were included at concentrations from 0 to 250 nM. In each well, 80 μL of concentrated reaction mix, 10 μL of MTDIA inhibitor solution, and 10 μL of lysed blood (corresponding to 0.125 μL blood per well) were added. Fluorescence was monitored for 1.5 h to capture both initial rate and slow-onset inhibition [21]. *K*_i_ values were determined from initial reaction rates for the first 800 s, while *K*_i_* values were obtained by comparing rates following slow-onset inhibition in the final seconds (4500 to 5000) and compared to the initial inhibitor-free rate. Inhibitor and control rates were then fit to the experimental inhibitor concentrations using the Morrison quadratic equations for tight-binding inhibitors (eq. (2) and (3)) [22].

(eq. 2) $$V_{0}^{'}=V_{0}\left(1-\left(\left(\left({\left[\text{E}\right]}_{\text{t}}+\left[\text{I}\right]+K_{\text{i}}^{*}\right)-{\left({\left([\text{E}{]}_{\text{t}}+[\text{I}{]}_{\text{t}}+K_{\text{i}}^{*}\right)}^{2}-4[\text{E}{]}_{\text{t}}[\text{I}{]}_{\text{t}}\right)}^{0.5}\right)/\left(2{\left[\text{E}\right]}_{\text{t}}\right)\right)\right)$$

where

(eq. 3) $$K_{\text{i}}^{*}=\left(K_{\text{i}}\left(1+\left(\left[\text{S}\right]/K_{\text{m}}\right)\right)\right)$$

and *V*′_0_ and *V*_0_ are the rates in the presence and absence of inhibitor, respectively, [*E*] is the enzyme concentration, [*I*] is the inhibitor concentration, [*S*] (1.5 mM MTA) is the substrate concentration, and *K*_m_ is the Michaelis constant for MTA (*K*_m_ = 4.2 μM).

### 2.8. MTAP titration in blood

Fresh whole blood from non-cancer and from cancer-diagnosed donors was lysed to a concentration of 1.25% (v/v) and incubated with varying concentrations of *p*ClPhTDIA for 2 h at room temperature. After incubation, 10 μL of the treated lysate was added to a reaction mixture containing 100 mM K_2_HPO_4_ (pH 7.4), 0.1 U/mL xanthine oxidase, 0.1 U/mL horseradish peroxidase, 0.5 μM adenine deaminase, 40 μM Amplex Red, and 50 μM MTA, corresponding to a final lysed blood concentration of 0.125 μL/well.

### 2.9. MTAP in routine-admission and cancer-diagnosed bloods

Reactions were conducted in 100 mM K_2_HPO_4_ (pH 7.4), 500 μM MTA, 0.1 U/mL horseradish peroxidase, 0.1 U/mL xanthine oxidase, 0.5 μM adenine deaminase, and 40 μM Amplex Red. Each reaction was initiated by adding 10 μL of fresh, unfrozen lysed blood to 90 μL of master mix, corresponding to the catalytic activity from 0.125% of whole blood. The assay was performed with blood from 28 routine-admission and 19 cancer-diagnosed clinical blood samples to compare MTAP activity.

### 2.10. MTAP activity in cancer cell lines

Cells were harvested with 0.05% trypsin, pelleted by centrifugation, washed in complete medium, and resuspended in 0.1% Triton X-100 prepared in 100 mM K_2_HPO_4_ (pH 7.4). Lysates were incubated on ice for 45 min to achieve complete lysis, then centrifuged at 14,000 rpm for 10 min at 4 °C to remove insoluble debris. The resulting supernatant was collected, and total protein concentration was quantified using the BCA assay.

Assays contained 100 mM K_2_HPO_4_ buffer (pH 7.4), 500 μM MTA, 0.1 U/mL horseradish peroxidase, 0.1 U/mL xanthine oxidase, 0.5 μM adenine deaminase, and 40 μM Amplex Red. Reactions were initiated by adding 10 μL lysate. Initial reaction rates were calculated from the linear phase of product formation. Controls without MTA were used to correct background signals arising from lysate components. Subsequent comparative analysis between cancer cell lines were performed at 5 μg/mL total protein (0.5 μg/100 μL assay chamber).

### 2.11. Western blots

Cultured cancer cells were lysed in 100 μL of RIPA buffer (Boston Bioproducts NC9193720) supplemented with Protease Inhibitor Cocktail (Sigma P8340), Phosphatase Inhibitor Cocktail 2 (Sigma P5726), and Phosphatase Inhibitor Cocktail C3 (Sigma P0044). Lysates were incubated on ice for 10 min and then sonicated (Fisher 422-A) using 5-s on/5-s off cycles for a total of 15 s on-time at 20% amplitude. Cleared lysates were mixed with 4 × Laemmli sample buffer (Bio-Rad 1610747) containing 2-mercaptoethanol (Sigma M7522) and heated at 85 °C for 10 min. Final lysates were loaded onto 4–20% Mini-PROTEAN^®^ TGX^™^ gels (Bio-Rad 4561096) alongside the Precision Plus Protein^™^ Dual Color Standards ladder (Bio-Rad 1610374). Electrophoresis was performed using 1 × running buffer (Bio-Rad 1610772) at 160 V for 35–40 min. Proteins were transferred to activated PVDF membranes (Millipore IPVH00010) using a TransBlot Turbo system (Bio-Rad 1704150) with 1 × transfer buffer (Bio-Rad 1610771), methanol (Fisher A452SK4), and 10% SDS (Lonza 51213).

Membranes were blocked for 1 h in 1 × TBS (Bio-Rad 1706435) containing Tween 20 (Fisher T0543500G) (TBST) supplemented with 10% milk (Bio-Rad 1706404). After blocking, membranes were incubated with primary antibody diluted 1:1000 in TBST with 10% milk overnight at 4 °C with gentle rocking. Primary antibodies were rabbit anti-MTAP (Abcam ab96231) and mouse anti-β-actin (Cell Signaling Technology, #3700). Membranes were then washed three times with TBST for 6 min per wash, followed by incubation with HRP-conjugated secondary antibody, against mouse (Invitrogen #32430) or rabbit (BioLegend #406401), diluted 1:5000 in TBST with 10% milk for 1 h at room temperature. Freshly prepared ECL solution (Bio-Rad 1705061) was applied to the membranes, and signal was detected using a Li-Cor Odyssey FC imaging system (Li-Cor Biosciences).

## 3. Results and discussion –

### 3.1. Optimization of assay conditions

MTAP catalytic activity produces a fluorescent spectral change in this coupled enzyme assay based on its adenine product. Adenine is deaminated by adenine deaminase (*Pa*ADA) to form hypoxanthine. Xanthine oxidase (XanOx) oxidizes hypoxanthine in two H_2_O_2_-generating steps to form uric acid. Hydrogen peroxide reacts with Amplex Red (ADHP) in the presence of horseradish peroxidase (HRP) to produce resorufin, a chromophore with 510 nm excitation and 590 nm fluorescence emission maxima. For each equivalent of adenine, two equivalents of hydrogen peroxide are produced (Scheme 1). This fluorescence detection method is rapid, compatible with 96-well high-throughput formats, and suitable for applications where small sample volume and turnaround time are critical.

Hydrogen peroxide standard curves ranging from 39 nM to 10 μM were generated under assay conditions containing XanOx, HRP, *Pa*ADA, recombinant *hs*MTAP, and ADHP in 100 mM K_2_HPO_4_ at pH 7.4, resulting in linear fluorescence responses. The observed response was 8.9 ± 0.2 × 10^5^ RFU per μM H_2_O_2_ (R^2^ = 0.998) (Fig. 1A). When the same calibration was conducted with 0.5 μg of cancer cell lysates, the resulting slope was 8.7 ± 0.4 × 10^5^ RFU per μM H_2_O_2_ (R^2^ = 0.971), which closely matched the purified MTAP conditions. This result demonstrates that the lysate matrix does not interfere with the fluorescent readout (Fig. 1A). As a result, raw fluorescence rates (ΔRFU/s) were converted to reaction rates (μM/s) with either calibration slope, allowing for direct quantification of hydrogen peroxide formation in subsequent assays.

Titrations of recombinant *hs*MTAP were performed under increasing coupled-enzyme conditions to establish conditions where coupling enzymes did not limit the initial reaction rates. A linear response to purified *hs*MTAP concentrations was established in the presence of 0.5 μM *Pa*ADA, 0.1 U/mL XanOx, 0.1 U/mL HRP, 40 μM ADHP, with 100 mM K_2_HPO_4_ at pH 7.4. MTAP concentrations from 0.01 to 4 nM gave a linear response (R^2^ = 0.999) (Fig. 1B). Fluorescence emission was quantified using the hydrogen peroxide standard curve from purified MTAP (Fig. 1A), thereby ensuring an accurate correlation between adenine production and signal output.

The assay conditions for measuring MTAP activity in human blood used whole blood lysed with 0.1% Triton X-100 in 100 mM K_2_HPO_4_ (pH 7.4). MTAP activity was assayed at increasing concentrations ranging from 0.01 to 1% relative to whole blood. Initial reaction rates (*v*_0_) increased proportionally with blood concentration up to 0.25% (Fig. 1C) (R^2^ = 0.965). At higher blood concentrations, the response was non-linear, because of the hemoglobin interference at both the 510 nm excitation and 590 nm emission wavelengths [23,24]. Based on these titrations, a final blood concentration of 0.125% (0.125 μL/well) was selected as it fell within the linear range and provided a robust assay signal-to-noise ratio (Fig. 1C).

Quantitation of MTAP activity in the presence of optical interference from hemoglobin was established using fluorescence signals from hydrogen peroxide standard curve titrations (4.5 nM to 11 μM) in reactions containing 0.125% lysed blood, corresponding to the conditions for MTAP assays. Fluorescence was a linear function of H_2_O_2_ to 11 μM but with a reduced slope of 3.9 ± 0.2 × 10^5^ RFU per μM (R^2^ = 0.992), quantitating the partial quenching by blood components (Fig. 1A). Linearity in activity response as a function of blood and *hs*MTAP dilutions assures that coupled enzyme activity is in excess.

### 3.2. MTAP activity in lysed human blood

**Fig. 2.**
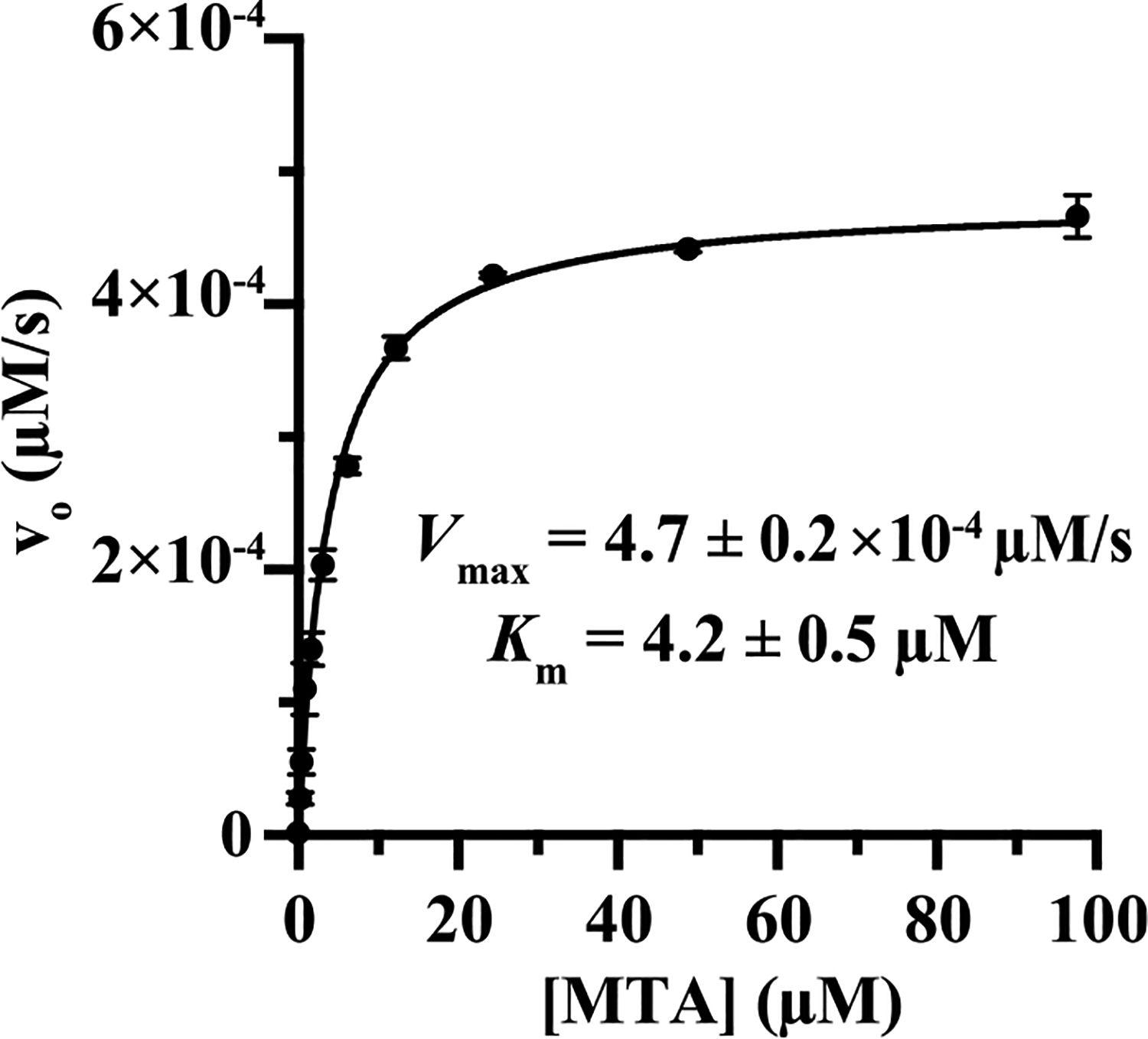
Michaelis-Menten kinetics of 5′-methylthioadenosine (MTA) phosphorolysis. Maximal velocity (*V*_max_) and the Michaelis constant (*K*_m_) for MTAP from human blood.

Initial reaction rates for MTAP in blood were measured as a function of substrate concentration using a titration of 5′-methylthioadenosine (MTA) under the 0.125 μL/well conditions described above. Background RFU increase in blood samples with no substrate relative to buffer only controls was indistinguishable, relating to a negligible free adenine concentration in the dilute blood assay. The lack of adenine interference agrees with previously reported concentrations of free adenine in whole blood, at 0.4 μM [25]. The lysed blood *hs*MTAP saturation curve with MTA demonstrated Michaelis-Menten kinetics, with a maximal reaction rate (*V*_max_) of 4.7 ± 0.2 × 10^−4^ μM/s/0.125 μL and a *K*_m_ of 4.2 μM ± 0.5 μM (Fig. 2). The Michaelis-Menten kinetics and *K*_m_ values are consistent with those previously reported for recombinant *hs*MTAP [26]. Both purified and blood-derived MTAP displayed similar kinetic properties and were not influenced by additional components present in lysed blood. Establishing these parameters under blood-based conditions enables the evaluation of MTAP inhibition *in vivo*, and for comparing inhibitor potency and residual activity in biological samples.

### 3.3. MTAP inhibition assays

**Fig. 3.**
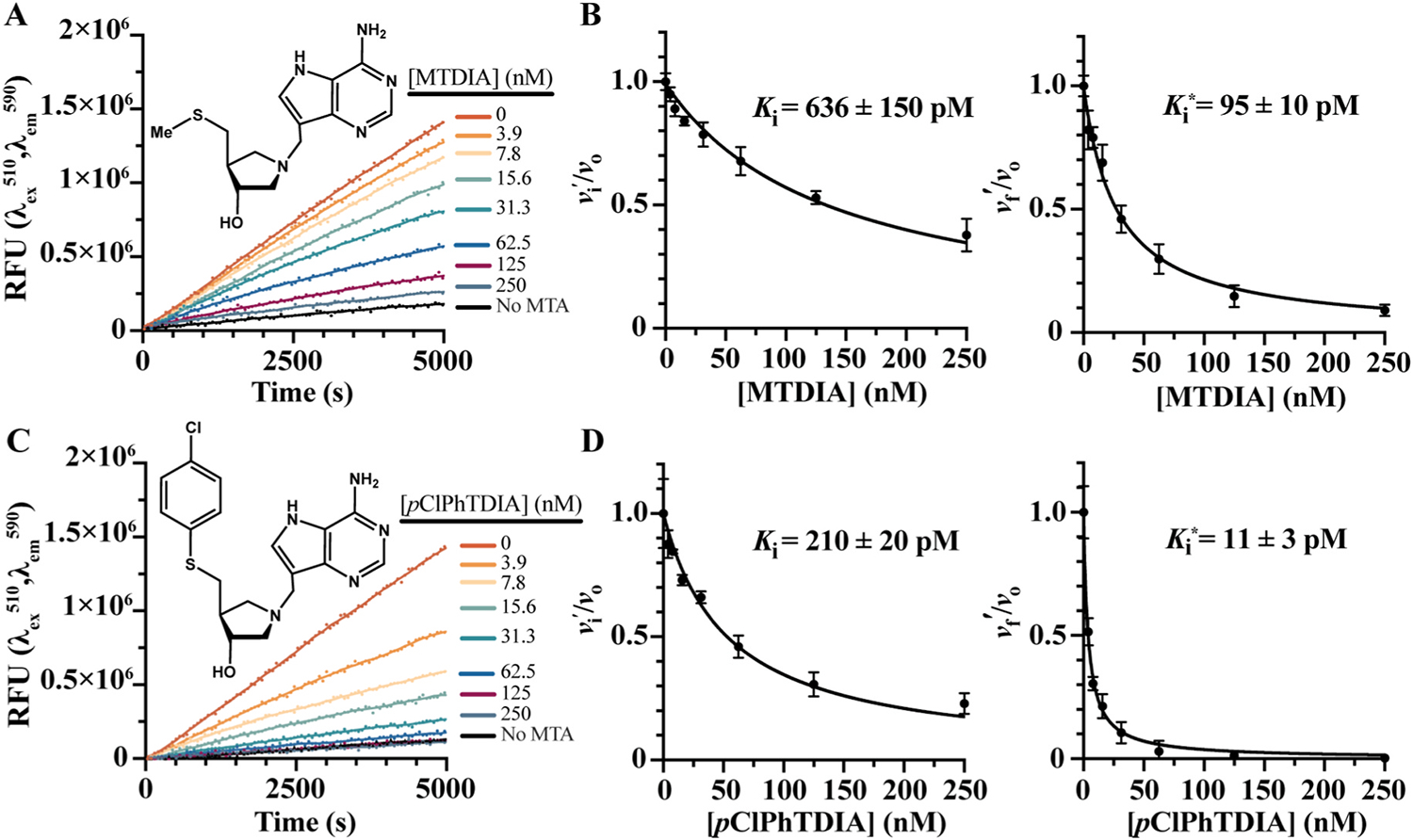
Inhibition of human blood MTAP by transition state analogs methylthio-DADMe-Immucillin-A (MTDIA) and *para*-Chloro-Phenylthio-DADMe-Immucillin-A (*p*ClPhTDIA). **(A)** Structure of MTDIA along with initial rates for *K*_i_ determination. **(B)** Values of *K*_i_ and *K*_i_* for MTDIA were obtained from initial rates and final rates. **(C)** Structure of *p*ClPhTDIA along with initial rates for *K*_i_ determination. **(D)** Values of *K*_i_ and *K*_i_* for *p*ClPhTDIA were obtained from initial rates and final rates.

Kinetic analyses of blood MTAP using the transition state analog inhibitors MTDIA and *p*ClPhTDIA (Fig. 3A–C) were used to evaluate the robustness of the human blood assay and to determine if blood *hs*MTAP is functionally equivalent to the enzyme expressed in *E. coli*. Using the coupled-enzyme assay conditions with 1.5 mM MTA as substrate, MTDIA yielded an apparent *K*ᵢ of 636 ± 150 pM from initial velocities and a tighter *K*ᵢ* of 95 ± 10 pM from final rates following time-dependent slow-onset equilibration (Fig. 3B). *p*ClPhTDIA exhibited an apparent *K*ᵢ of 210 ± 20 pM and *K*ᵢ* of 11 ± 3 pM, reflecting the reported enhanced affinity relative to MTDIA (Fig. 3D). Both MTDIA and *p*ClPhTDIA display the time-dependent onset of inhibition reaction curves characteristic of transition state analogs (Fig. 3A–C) [13].

The observed *K*ᵢ values for MTDIA and *p*ClPhTDIA are lower than previously reported, a consequence of the short lag in obtaining steady-state rates from the multiple coupling enzyme system. During this time, a fraction of the slow-onset phase is initiated. In contrast, *K*ᵢ* values are measured at longer time intervals following full enzyme-inhibitor equilibration, a condition this assay inherently achieves. The concordance between the measured *K*ᵢ* values and published data for recombinant *hs*MTAP confirms that enzymes from both sources exhibit similar responses to inhibitors and that the assay accurately reproduces physiologically relevant transition state binding interactions.

### 3.4. Active MTAP concentration in blood

**Fig. 4.**
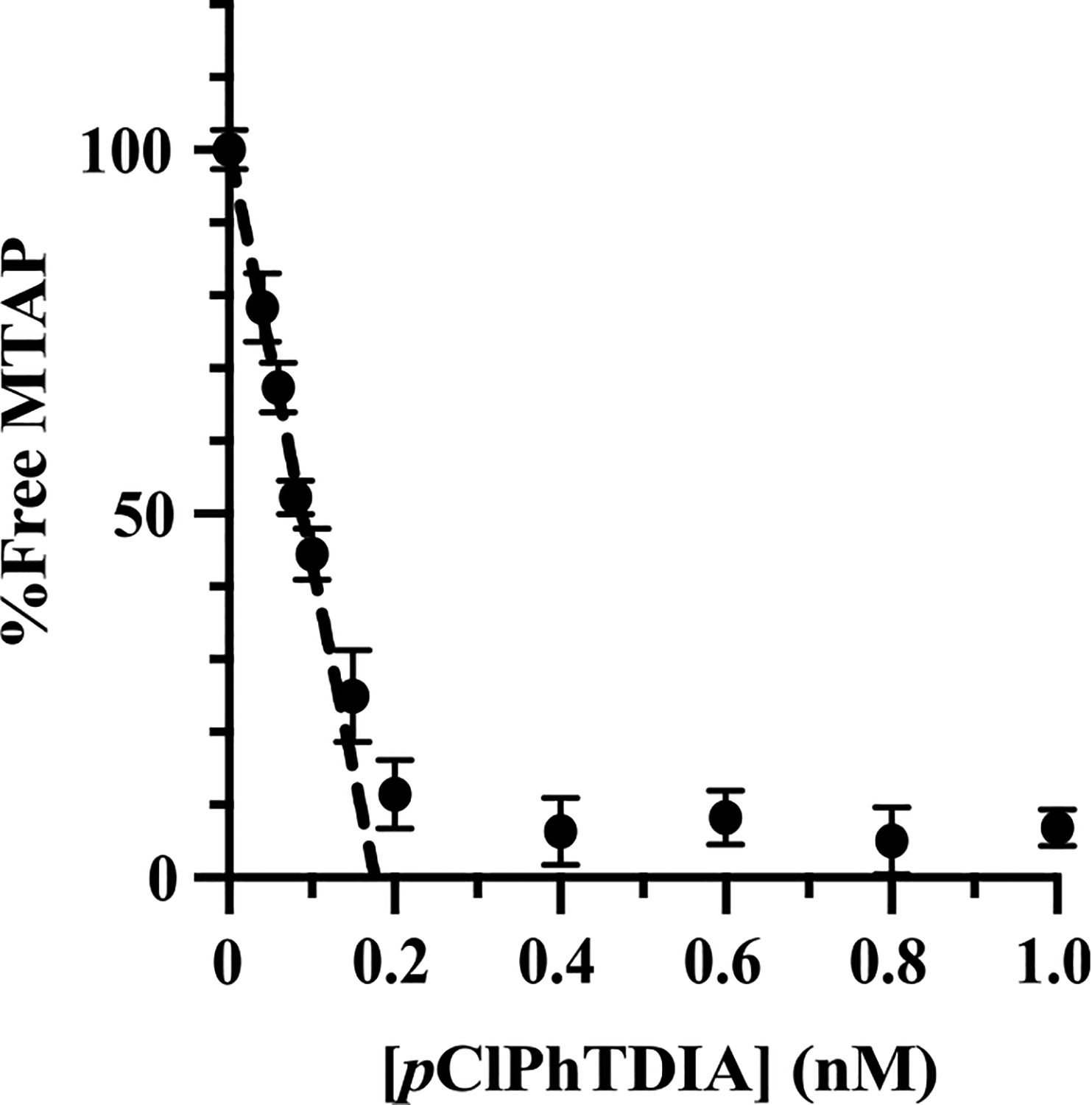
Catalytic activity titration of MTAP in 0.125% lysed human blood by inhibition with tight-binding inhibitor *p*ClPhTDIA.

Tight-binding enzyme inhibitors can be used to titrate catalytic activity and thereby determine the concentration of active enzymes in biological samples. Pooled blood was lysed as in the methods and incubated with *p*ClPhTDIA for 2 h to form the tightly bound complex. Samples were assayed to measure initial rates of product formation (Fig. 4). Using the 0.125% blood-in-well assay, catalytically active MTAP concentration was determined to be 0.176 ± 0.011 nM. Extrapolation to 100% whole blood yields an MTAP active concentration of 141 ± 9 nM.

### 3.5. MTAP from routine and cancer blood samples

**Fig. 5.**
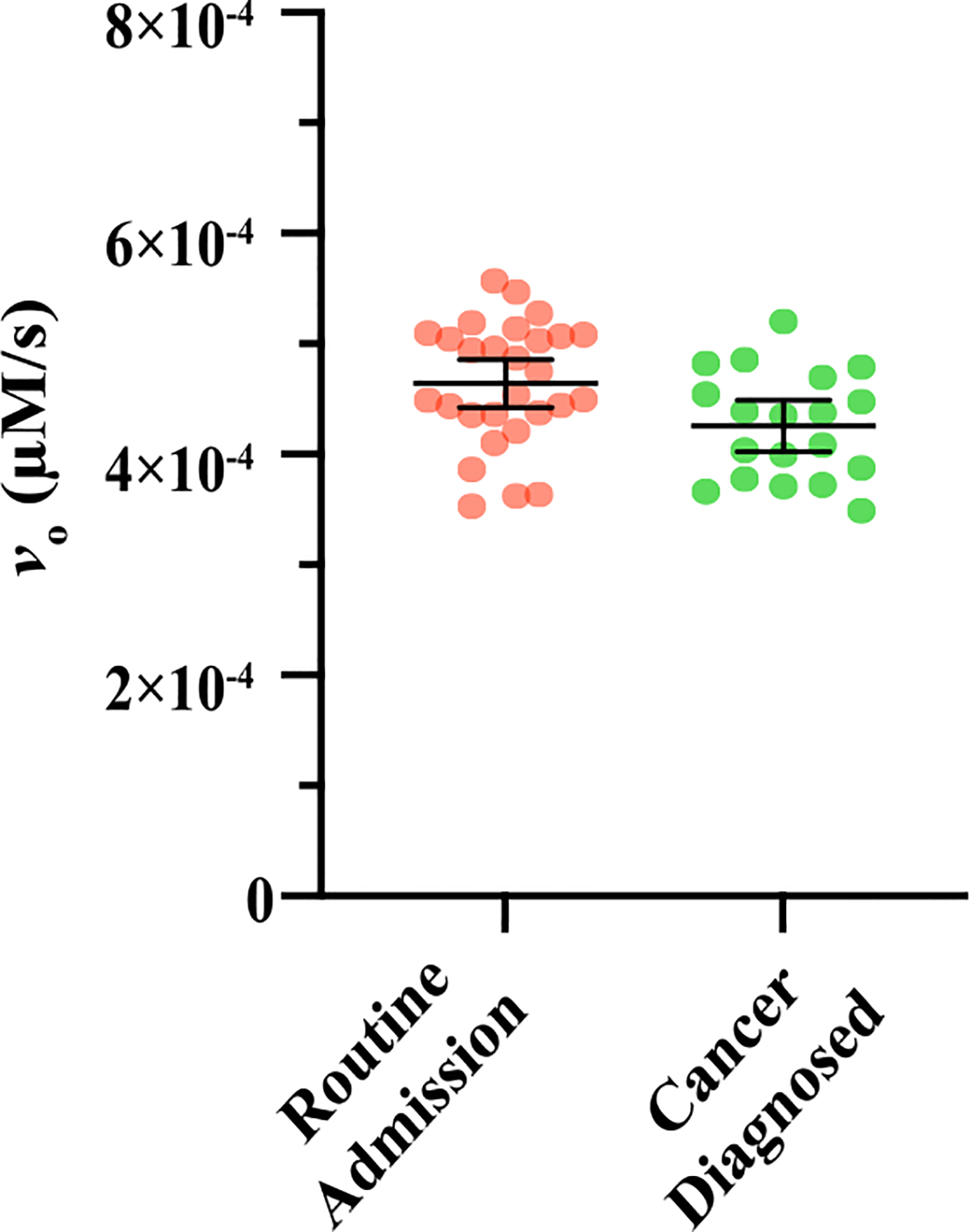
Initial rates of MTAP activity from lysed human blood samples in 28 routine-admission and 19 cancer-diagnosed patient samples. Each point represented is an average of n = 3 replicate experiments per donated blood sample.

Blood samples from 28 routine-admission donors and 19 cancer-diagnosed donors were analyzed to estimate an average MTAP activity from these populations. At 0.125 μL/well, the average MTAP activities measured were 4.6 ± 0.5 × 10^−4^ μM/s for routine-admission samples and 4.3 ± 0.1 × 10^−4^ μM/s for cancer-diagnosed samples (Fig. 5), a weakly significant difference in rates (p value of 0.016). The MTAP catalytic turnover numbers were similar within experimental error for both groups: 2.7 ± 0.3 s^−1^ (routine samples) and 2.5 ± 0.1 s^−1^ (cancer-diagnosed). These values are consistent with the *k*_cat_ published values obtained using fluorescent assays of purified MTAP activity [27]. When normalized to an average hemoglobin content for all deidentified samples, assuming 150 mg Hb/mL blood [28,29], the apparent specific activities were 8.8 ± 1.0 nmol mg^−1^ Hb^−1^ h^−1^ and 8.3 ± 0.2 nmol mg^−1^ Hb^−1^ h^−1^, respectively. These results are comparable to the previously reported MTAP activity of 8.9 ± 2.0 nmol mg^−1^ Hb^−1^ h^−1^, thereby validating sample preparation and assay conditions [15]. Considering the 800-fold dilution between whole blood and the 0.125% in-well concentration, the estimated whole-blood active MTAP concentration is approximately 140 nM.

The similarity in rate between routine-admission and cancer-diagnosed patient samples shows that MTAP activity is a reproducible measure across individuals. This consistency makes MTAP activity a reliable biomarker for monitoring inhibition by slow-onset, tight-binding inhibitors. Consequently, measuring MTAP activity during treatment is advantageous, as MTAP is the direct therapeutic target capable of inducing synthetic-lethal interactions when co-targeted with MAT2A or PRMT5 [9]. Establishing a consistent baseline of MTAP activity allows decreases in signal to be confidently attributed to inhibitor-mediated inhibition.

### 3.6. MTAP activity in cancer cell lines

**Fig. 6.**
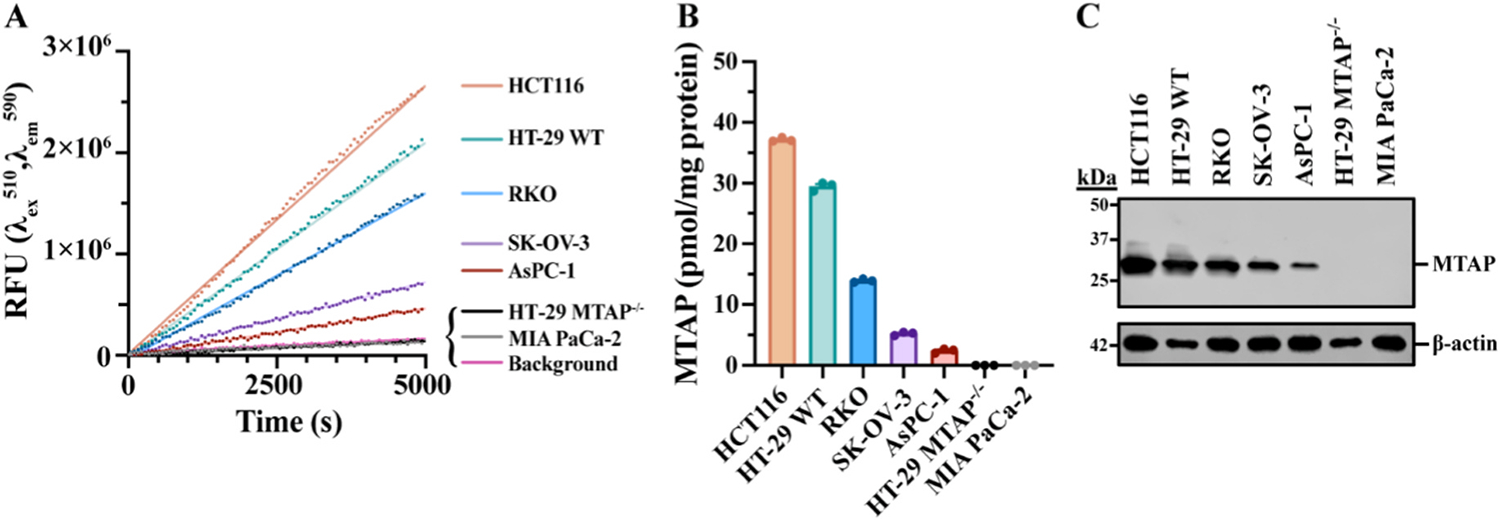
MTAP activity and expression in cancer cell lines. **(A)** Representative fluorescent traces showing MTAP activity in lysates (0.005 mg/mL protein). **(B)** MTAP activities (mean ± SD, pmol/mg protein) derived from initial rates. **(C)** Western blot showing MTAP expression, with β-actin as loading control. Data represented is an average of n = 3 replicate experiments per cancer cell line.

MTAP activity was quantified in several cancer cell-line lysates normalized by protein content. MTAP content was assessed using a range of lysate concentrations, and a linear response range was confirmed up to 5 μg/mL (HCT116, R^2^ = 0.995; HT29, R^2^ = 0.993; RKO, R^2^ = 0.999, SK-OV-3, R^2^ = 0.998, AsPC-1, R^2^ = 0.971). Free adenine detection in no substrate controls was negligible compared to background in cell lysate conditions over the course of kinetic reads, as described above for blood MTAP activity. Enzymatic activity varied significantly among the cancer cell lines. The colon cancer cell line HCT116 showed the highest activity at 37.2 ± 0.3 pmol/mg protein, followed by colon cancer line HT-29 at 29.4 ± 0.7 pmol/mg protein. The RKO colon cancer line displayed intermediate activity at 14 ± 0.3 pmol/mg protein. In contrast, the ovarian adenocarcinoma SK-OV-3 and metastatic pancreatic cancer AsPC-1 displayed lower activities at 5.2 ± 0.2 pmol/mg protein and 2.4 ± 0.3 pmol/mg protein, respectively. MTAP-null lines HT-29 MTAP^−/−^ and pancreatic cancer line MIA PaCa-2 showed no detectable activity, consistent with their genetic status, as a result, confirming assay specificity (Fig. 6A and B).

Cell lines that express MTAP showed immunoassay band intensities matching their measured activities. In contrast, *MTAP*-deleted HT-29 MTAP^−/−^ and MIA PaCa-2 had no detectable MTAP protein, with β-Actin serving as the loading control. The agreement between protein expression and enzymatic activity confirms the specificity of the assay and validates the observed distribution of MTAP activity across cancer cell lines (Fig. 6C).

### 3.7. Clinical relevance of MTAP analysis

Currently, more than a dozen clinical trials are underway for MTAP-null cancer, targeting either PRMT5 or MAT2A, both synthetic-lethal partners with MTAP deletion [30]. These programs focus on the 15% of cancer patients with MTAP^−/−^ solid tumors, leveraging the genetic synthetic lethality resulting from the overabundance of MTA and/or decrease in S-adenosylmethionine. The combination of *hs*MTAP inhibitors with PRMT5 or MAT2A agents is proposed to expand this synthetic lethal approach to MTAP^+/+^ cancers. The development of practical pharmacodynamic assays with rapid turnaround times is essential for monitoring responses to these emerging cancer therapies. The flexibility of the described assay enables assessment of cancer tissues, as demonstrated by the cancer cell line results. As tissue biopsy samples typically give samples approximately 8 mg of tissue, or 1600 μg of protein [31], the 0.5 μg per well sensitivity of this assay permits the facile use of biopsy material. This ultrasensitive MTAP assay therefore provides a predictive biomarker, enabling rapid identification of patients most likely to benefit from PRMT5 or MAT2A inhibitor therapies, and establish the *in vivo* effect of targeting *hs*MTAP with transition state analogs.

## 4. Conclusions –

We have developed a fluorescent assay that quantifies endogenous MTAP activity with high sensitivity in blood and tissues. This assay establishes similar kinetic constants for *hs*MTAP in whole blood to those for recombinant *hs*MTAP, confirming target specificity. This assay also demonstrates inhibition parameters for MTDIA and *p*ClPhTDIA that align with those obtained from purified enzyme studies. Baseline MTAP activities were similar in the cohorts of routine blood-sampled individuals and patients diagnosed with cancer, indicating that blood MTAP is a stable biomarker that allows high confidence in signal changes and provides good evidence for pharmacologic inhibition, with little patient-to-patient variability.

Profiling MTAP activity in cancer cells revealed a wide range of MTAP activity that closely matches MTAP protein levels. This alignment between activity and expression underscores the relevance of the assay to tumor biology and further supports its utility for tracking on-target MTAP inhibition *in vivo*.

By linking MTAP enzymology into a liquid or tumor biopsy strategy, this work establishes a foundation for real-time pharmacodynamic monitoring in clinical trials and to guide adaptive treatment that exploits MTAP-PRMT5 or MTAP-MAT2A synthetic lethality [9,12,30,32]. In doing so, MTAP activity serves as both a direct indicator of drug action and a practical biomarker to inform patient personalized cancer therapies targeting metabolic vulnerabilities.

## Data Availability

Data will be made available on request.

## Abbreviations

MTAP: Methylthioadenosine phosphorylase
PRMT5: Protein arginine methyltransferase 5
MAT2A: Methionine adenosyltransferase 2A
MTA: 5′-methylthioadenosine
XanOx: Xanthine oxidase
*Pa*ADA: *Pseudomonas aeruginosa* Adenine deaminase
HRP: Horseradish peroxidase
MTDIA: Methylthioadenosine-DADMe-Immucillin-A
*p*ClPhTDIA: *para*-Chloro-Phenylthio-DADMe-Immucillin-A
